# Gaucher or pseudo-Gaucher cells

**DOI:** 10.1007/s44313-024-00005-x

**Published:** 2024-02-28

**Authors:** Gurpreet Kaur, Ankur Ahuja, Ganesh Kumar Vishwananthan, Arijit Sen

**Affiliations:** Department of Hematology, All India Institute of Medical Sciences, Ansari Nagar East, New Delhi, 110049 India

**Fig. 1:**
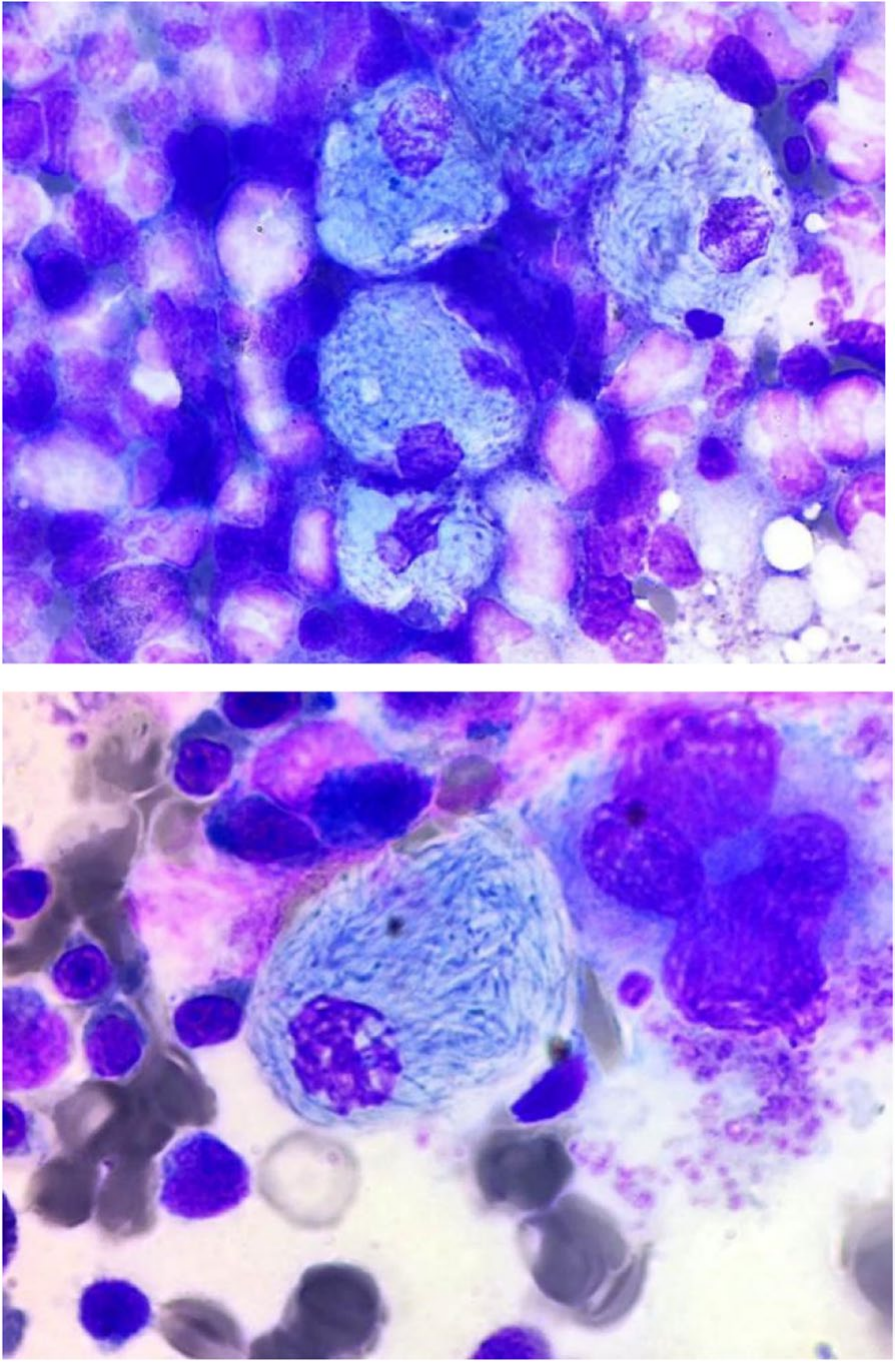
Aggregates of Pseudo-gaucher or histiocytes with cells with rounded, blue, lamellar cytoplasm resembling "onion skin" or "crumpled tissue paper"

The patient, a 65-year-old man, presented with a prolonged history of upper abdominal discomfort, anorexia, and weight loss. Examination revealed splenomegaly 15 cm below the left costal margin. There was leukocytosis with moderate anemia. Bone marrow examination revealed hypercellular marrow with a myelogram that showed: myeloblasts 02%; promyelocytes 02%; myelocytes 28%; metamyelocytes 17%; band forms 20%; neutrophils 20%; and basophils 5%, eosinophils 03%, lymphocytes 03%. There was a mild increase in megakaryocyte number with occasional dwarf forms, and numerous pseudo-Gaucher cells with a crumpled tissue paper appearance were seen. Reverse transcription-PCR for BCR–Abl was positive for p210 transcript. Pseudo-Gaucher cells occur in a variety of conditions, such as sickle cell anemia, congenital dyserythropoietic anemias, immune thrombocytopenia, acute lymphoblastic leukemia, multiple myeloma, myelodysplasia, Hodgkin’s disease, thalassemia, and disseminated mycobacterial infection. Pseudo-Gaucher cells cannot be distinguished from true Gaucher cells by routine hematoxylin–eosin staining; however, iron stain can be used to differentiate them, in which case Gaucher cells exhibit diffuse iron staining whereas pseudo-Gaucher cells do not. Electron microscopy shows that Gaucher cells contain typical tubular cytoplasmic inclusions absent in pseudo-Gaucher cells. The knowledge of possible associations, appropriate immunohistochemistry, and relevant additional investigations are necessary for final diagnosis (Fig. 1).

